# Predicting physical function and mental health in trauma intensive care patients 2 years after hospitalisation

**DOI:** 10.1186/cc12420

**Published:** 2013-03-19

**Authors:** L Aitken, B Macfarlane, W Chaboyer, M Schuetz, C Joyce, AG Barnett

**Affiliations:** 1 Princess Alexandra Hospital, Brisbane, Australia; 2 Griffith University, GoldCoast, Australia; 3 Queensland University of Technology, Brisbane, Australia

## Introduction

Long-term compromise after traumatic injury is significant; however, few modifiable factors that influence outcome have been identified. The aim of this study was to identify acute and early post-acute predictors of long-term recovery amenable to change through intervention.

## Methods

Adults (>17 years) admitted to the ICU, Princess Alexandra Hospital, Australia following injury were prospectively followed. Data were collected on demographics, pre-injury health, injury characteristics and acute care factors. Psychosocial measures (self-efficacy (SE), illness perception (IP), post-traumatic stress disorder (PTSD) symptoms and psychological distress) and health status (SF-36) were collected via questionnaire 1, 6, 12, and 24 months post injury. Outcomes of interest were the Physical Function (PF) and Mental Health (MH) subscales of the SF-36. Regression models were used to estimate predictors of physical function and mental health over a 2-year period. A subject-specific intercept in a mixed model was used to account for repeated data from participants over time.

## Results

Participants (*n *= 123) were young (median 37, IQR 22 to 55 years), predominantly male (83%) and spent on average 3 days in the ICU and 3 weeks in hospital. Response rates were over 55% at each follow-up, with responders similar to nonresponders except for being generally older. PF and MH scores improved over time, although the averages remained below the Australian norms at 24 months. Predictors of PF included IP (β = -1.5, 95% CI = -3.1 to -1.1, *P *0.01), SE (β = 1.8, 95% CI = 1.3 to 2.6, *P *0.01), hospital length of stay (β = -1.7, 95% CI = -2.0 to -0.8, *P *0.01), never having been married (β = 1.8, 95% CI = 0.3 to 5.5, *P *= 0.03), and having injury insurance (β = -2.7, 95% CI = -6.9 to -1.9, *P *0.01). Predictors of MH included PTSD symptoms (β = -2.4, 95% CI -3.4 to -1.4, *P *0.01), psychological distress (β = -6.9, 95% CI = -8.9 to -5.2, *P *0.01), SE (β = 0.6, 95% CI = 0.2 to 1.1, *P *0.01), and unemployment (β = -2.3, 95% CI = -5.0 to -0.2, *P *= 0.04).

## Conclusion

Trauma ICU patients experience compromised physical function and mental health 24 months after injury. Psychological distress, self-efficacy and illness perception influence outcomes and are potentially amenable to change in response to interventions initiated during hospital stay.

